# Anatomy of ovary and ovule in dandelions (*Taraxacum*, Asteraceae)

**DOI:** 10.1007/s00709-012-0455-x

**Published:** 2012-09-23

**Authors:** K. Musiał, B. J. Płachno, P. Świątek, J. Marciniuk

**Affiliations:** 1 Department of Plant Cytology and Embryology, Jagiellonian University, Grodzka 52, 31-044 Krakow, Poland; 2 Department of Animal Histology and Embryology, University of Silesia, Bankowa 9, 40-007 Katowice, Poland; 3 Department of Botany, Siedlce University of Natural Sciences and Humanities, ul. Prusa 12, 08-110 Siedlce, Poland

**Keywords:** Apomictic plant, Ovule structure, Cell wall modification, Dandelion, *Taraxacum*, Asteraceae

## Abstract

The genus *Taraxacum* Wigg. (Asteraceae) forms a polyploid complex within which there are strong links between the ploidy level and the mode of reproduction. Diploids are obligate sexual, whereas polyploids are usually apomictic. The paper reports on a comparative study of the ovary and especially the ovule anatomy in the diploid dandelion *T. linearisquameum* and the triploid *T*. *gentile*. Observations with light and electron microscopy revealed no essential differences in the anatomy of both the ovary and ovule in the examined species. Dandelion ovules are anatropous, unitegmic and tenuinucellate. In both sexual and apomictic species, a zonal differentiation of the integument is characteristic of the ovule. In the integumentary layers situated next to the endothelium, the cell walls are extremely thick and PAS positive. Data obtained from TEM indicate that these special walls have an open spongy structure and their cytoplasm shows evidence of gradual degeneration. Increased deposition of wall material in the integumentary cells surrounding the endothelium takes place especially around the chalazal pole of the embryo sac as well as around the central cell. In contrast, the integumentary cells surrounding the micropylar region have thin walls and exhibit a high metabolic activity. The role of the thick-walled integumentary layers in the dandelion ovule is discussed. We also consider whether this may be a feature of taxonomic importance.

## Introduction

The Asteraceae (Compositae) family is one of the largest flowering plant families. With over 24,000 recognized species, this family constitutes ca. 10 % of all flowering plants (Funk et al. 2009). Asteraceae is usually divided into three subfamilies: (1) the small South American Barnadesioideae, which contains less than 1 % of the species, (2) the Asteroideae, which contains ca. 65 % of the species and (3) the Cichorioideae (syn. Lactucoideae), which comprises ca. 35 % of the species (Bremer et al. 1992; Funk et al. 2009). However, it should be noted that according to a new higher classification system there are 12 subfamilies within Asteraceae (Funk et al. 2009). Analyses based on both morphological and molecular data show that the subfamilies Barnadesioideae and Asteroideae are monophyletic, whereas the status of Cichorioideae remains uncertain although molecular findings strongly support the monophyly of this subfamily; however, the morphological data indicate that Cichorioideae is most likely paraphyletic (Bremer et al. 1992; Anderberg et al. 2007; Funk et al. 2009).

*Taraxacum* Wigg. (dandelions) is a very large cosmopolitan genus belonging to the subfamily Cichorioideae, the tribe Cichorieae and the subtribe Crepidinae (Anderberg et al. 2007; Kilian et al. 2009). *Taraxacum* comprises both diploid (2*n* = 2*x* = 16) and polyploid species, mainly triploids (2*n* = 3*x* = 24) or tetraploids (2*n* = 4*x* = 32), but higher ploidy levels have also been reported (Kirschner and Štěpánek 1996). It is well known that in angiosperms, polyploidy is closely connected with apomixis (Hörandl 2010). In *Taraxacum*, moreover, there is a correlation between the ploidy level and the mode of reproduction. The great majority of dandelions are polyploids and obligate or facultative agamosperms that produce seeds asexually, whereas rarely occurring diploid species reproduce sexually (Richards 1973, 1989). Apomixis in *Taraxacum* includes three elements — meiotic diplospory (Gustafsson 1946; Nogler 1984; Asker and Jerling 1992), parthenogenesis and autonomous endosperm formation (Richards 1973). The co-occurrence of apomixis and sexual reproduction is the main evidence that *Taraxacum* is an aggregate of a large number of species and that its taxonomy is very complicated (Kirschner et al. 2003). The infrageneric classification of this taxon is based on the sectional concept (Kirschner and Štěpánek 1997). The genus is divided into over 55 sections comprising about 3,000 species and new species are still being described (Záveská Drábková et al. 2009; Mártonfiová et al. 2010; Uhlemann 2010; Marciniuk et al. 2012).

Numerous species of *Taraxacum* from Europe have been studied embryologically (Małecka 1971, 1973 and references therein; van Baarlen et al. 2000, 2002). These investigations were focused on the analysis of female and male meiosis, on embryo development and endosperm formation, whereas the ovule structure has not received sufficient attention. As it is well known, ovules are the precursors of seeds and play an essential role in sexual as well as in apomictic reproduction of angiosperms. Currently, the results of classical and molecular genetic analysis of ovule development indicate that the sporophytic tissues of the ovule influence female gametophyte development, guidance of pollen tube growth, fertilisation, embryogenesis and finally seed formation (for a review, see Gasser et al. 1998; Skinner et al. 2004; Endress 2011). Moreover, the papers cited point out that ovules are very desirable structures for the study of the regulation of morphogenesis; thus, an understanding of ovule ontogeny will be helpful in elucidating various aspects of plant development. It should also be emphasized that certain ovule features are relatively conservative in evolution and have a very important taxonomic significance (Bouman 1984; Endress 2011).

To date, research data on details of the anatomy of the dandelion ovule are missing. Furthermore, it is worth mentioning that the ultrastructure of the ovule in the Asteraceae family has rarely been investigated, e.g., the most detailed study is about the model crop *Helianthus* (Newcomb 1973a, b; Yan et al. 1991) although *Cynara cardunculus* has also been investigated (Figueiredo et al. 2006).

The first aim of this study was to perform a structural description of dandelion ovules based on observations with light and electron microscopy. The results obtained would be the basis for further analyses of ovule structure within the tribe Cichorieae. A comparison of ovule anatomy in different species may provide useful data for taxonomical and phylogenetical investigations not only within the subfamily Cichorioideae but also for the entire Asteraceae.

The second aim of this study was to check whether there are differences in the ovule structure between sexual and apomictic *Taraxacum* species. We analysed the anatomy of the ovules in two *Taraxacum* species from the most common section *Ruderalia* that differ in ploidy level and reproduction mode: sexual *T*. *linearisquameum* Soest (2*n* = 2*x* = 16) and apomictic *T*. *gentile* G.E. Haglund & Rail. (2*n* = 3*x* = 24) (Góralski et al. 2009; http://www.binoz.uj.edu.pl).

## Materials and methods

### Plant material

Inflorescens of *Taraxacum gentile* were collected from a natural population in Chojniki near Baranowo in Poland (53°07′42″N, 21°23′14″E). Flowers of *T*. *linearisquameum* were sampled from specimens growing in the private collection of J. Marciniuk in Siedlce (52°10′49″N, 22°18′26″E); these plants were obtained from seeds collected by dr. R. Vašut in Moravian Silesia in the Czech Republic. Studies were carried out on capitula just before anthesis.

### Methods

#### Light and electron microscopy studies

For clearing technique, inflorescences were fixed in FAA (40 % formalin/glacial acetic acid/70 % ethanol, 5:5:90, v/v) for 24 h and stored in 70 % ethanol. Than isolated ovaries were dehydrated for 1 h in 70 %, 80 %, 90 % ethanol (one change) and 100 % ethanol (three changes), and incubated (for 1.5 h) in one change of ethanol/methyl salicylate (1:1), one change of ethanol/methyl salicylate (1:3) and two changes of 100 % methyl salicylate (Young et al. 1979; Musiał et al. 2012). Cleared ovaries were examined with a Nikon Eclipse 80i microscope equipped with Nomarski interference contrast optics.

The procedure for preparing samples for TEM was as described earlier (Płachno and Świątek 2009, 2010). Briefly, for the electron microscopy studies, ovaries were fixed in 2.5 % formaldehyde and 2.5 % glutaraldehyde in a 0.05 M cacodylate buffer (pH 7.0) for 2 days. The material was postfixed in 1 % OsO_4_ in a cacodylate buffer for 24 h at ~4 °C, rinsed in the same buffer, treated with 1 % uranyl acetate in distilled water for 1 h, dehydrated with acetone and embedded in an Epoxy Embedding Medium Kit (Fluka). Semithin sections were stained with methylene blue and examined using an Olympus BX60 microscope. The periodic acid-Schiff (PAS) reaction was used for visualisation of the total carbohydrates of insoluble polysaccharides (Wędzony 1996). Additionally, material embedded in paraffin (Musiał et al., 2012) or in Technovit 7100 (Kulzer, Germany) (Popielarska-Konieczna et al. 2012) was also used for (PAS) reaction. All results were the same: total carbohydrates of insoluble polysaccharides stain magenta to purplish red.

Ultrathin sections were cut on a Leica ultracut UCT ultramicrotome. After contrasting with uranyl acetate and lead citrate, the sections were examined using a Hitachi H500 electron microscope at 75 kV.

## Results

**Fig. 1:**
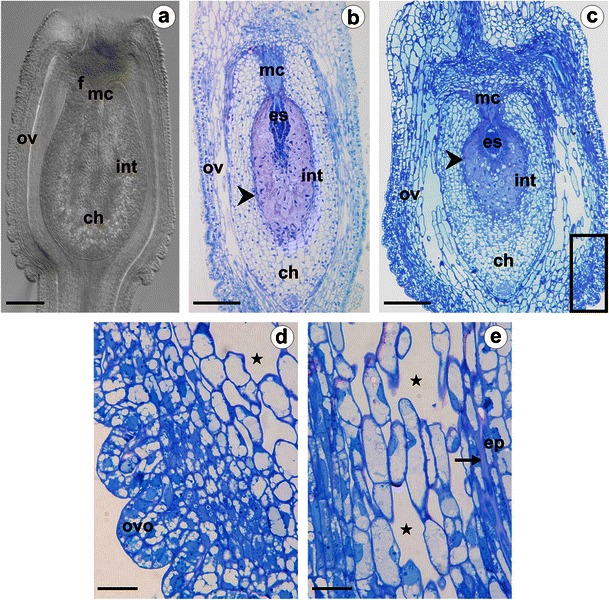
Anatomy of the ovary and ovule of *Taraxacum linearisquameum* (**a, b**) and *T*. *gentile* (**c**–**e**). **a** Ovary with anatropous, unitegmic, tenuinucellate ovule; image was obtained from unstained, cleared material using Nomarski DIC optics. **b, c** Median longitudinal sections through the ovaries and ovules; *arrowheads* indicate layers of thick-walled integumentary cells; *framed part* is shown in panel **d**. **d** Semithin section through the outer part of the ovary wall in the apical region; outgrowths (*ovo*) visible on the surface. **e** Semithin section through the inner part of the ovary wall. *ch* chalaza, *ep* outer epidermis of integument, *es* embryo sac, *f* funiculus, *int* integument, *mc* micropylar canal, *ov* ovary wall, *arrow* cuticle, *stars* intercellular spaces. *Bars*: **a**–**c** 100 μm; **d**, **e** 20 μm

**Fig. 2:**
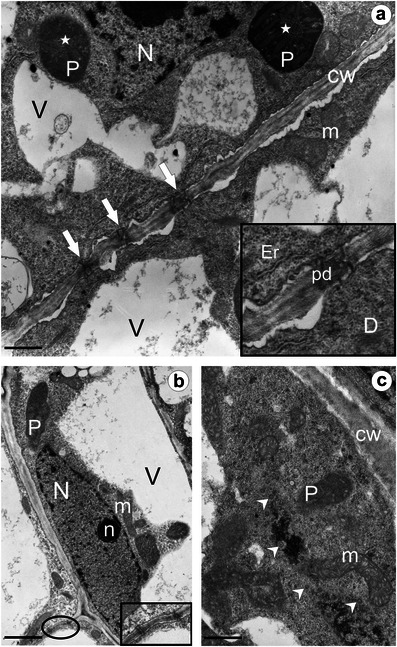
Ultrastructure of the ovary wall cells of *T*. *gentile*. **a** Section through an epidermal cell and a subepidermal parenchyma cell; *arrows* show branched plasmodesmata, *insert* branched plasmodesma at a higher magnification. **b** Fragment of parenchyma cells within the inner zone of the ovary wall; *elipse* plasmodesmata (*insert*) at a higher magnification. **c** Section through a parenchyma cell from the inner part of the ovary wall showing a dense cytoplasm rich in organelles. *Arrowheads* pores in nuclear envelope, *CW* cell wall, *D* dictyosome, *Er* endoplasmic reticulum, *m* mitochondrion, *N* nucleus, *n* nucleolus, *P* plastid, *pd* plasmodesma, *stars* prolammellar bodies, *V* vacuole. *Bars*: **a** 0.75 μm, **b** 1.5 μm, **c** 0.625 μm

Florets of dandelion species possess an inferior and unilocular ovary with a single ovule on the basal placenta (Fig. 1a). The ovaries as well as the ovules of the species studied differ slightly in shape. In *T*. *linearisquameum*, the ovary and the ovule are more elongated than in *T*. *gentile* (Fig. 1a–c). The surface of the ovaries is smooth in the basal and central part, while it is pleated in the apical region (Fig. 1a–d). Visible outgrowths form spikes on the surface of the achene. The mature ovule is anatropous, unitegmic and tenuinucellate (Fig. 1a) as it is known in other Asteraceae. Any notorious differences appeared in the anatomy of either the ovary or the ovule in the species examined. The ovary wall shows a zonal differentiation, which is particularly visible in the central part (Fig. 1b, c). The cells of the outer layers are smaller and closely packed, whereas, the ones of the inner zone are elongated, highly vacuolated and loosely arranged, parallel to the ovary axis (Fig. 1d, e). A single-layer epidermis is formed by vacuolated cells in which the nucleus is usually located in the basal part of the cell and the plastids, including prolammellar bodies, are clearly visible in TEM (Fig. 2a). The emphasised external walls of the epidermal cells are thickened and contain an electron-dense, thin layer of cuticle. Numerous branched plasmodesmata are present in the inner walls of the epidermis and the subepidermal parenchyma cells (Fig. 2a). Within the inner zone of the ovary wall, the cytoplasm of the cells is rich in ribosomes and mitochondria with well-developed cristae (Fig. 2b, c). There are also plasmodesmata in the cell walls (Fig. 2b).

**Fig. 3:**
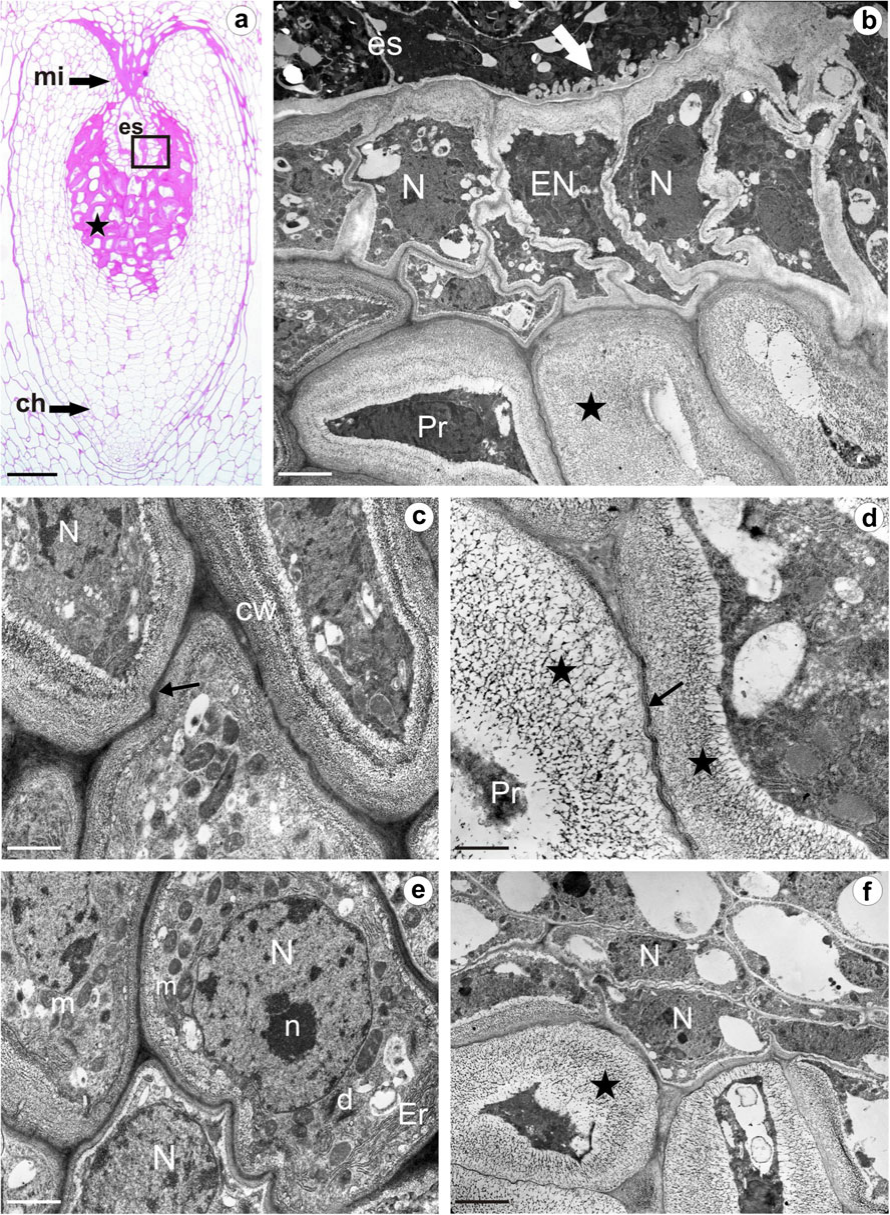
Anatomy and ultrastructure of *T*. *gentile* ovule; semithin section (**a**), electron micrographs (**b–f**). **a** Ovule after PAS reaction, note PAS-positive extremely thick walls (*star*) of the integumentary cells in the layers adjacent to embryo sac (*es*); *ch* chalazal pole, *mi* micropylar pole, *framed part* shown in panel **b**. **b** Section through the endothelium surrounding the embryo sac and thick-walled integumentary cells; *arrow* ingrowths on the wall of the central cell (*cc*), *EN* endothelium, *N* nucleus, *Pr* remains of protoplast, *star* prominent thick cell wall. **c** Ultrastructure of integumentary cells in the chalazal region of the embryo sac; *CW* cell wall, *N* nucleus, *arrow* middle layer. **d** Thick-walled integumentary cells, note the spongy structure of the walls (*stars*), remains of the protoplast (*Pr*) and waving middle lamella (*arrow*) between prominent thick cell walls. **e** Ultrastructure of integumentary cells in direct contact with the micropylar canal; *d* dictyosome, *Er* endoplasmic reticulum, *m* mitochondrion, *N* nucleus, *n* nucleolus. **f** Section through the thin-walled integumentary cells situated on the outside of the thick-walled zone; *N* nucleus, *star* extremely thick wall. *Bars*: **a** 50 μm, **b** 2.6 μm, **c–e** 1 μm, **f** 2 μm

**Fig. 4:**
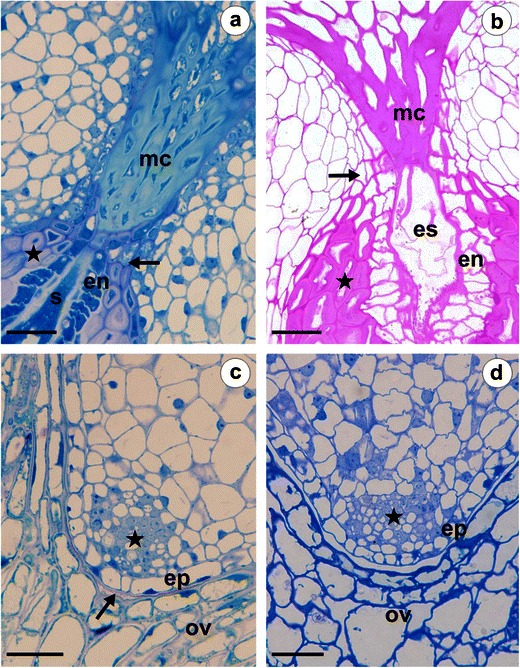
Anatomy of the ovule of *T*. *linearisquameum* (**a, c**) and *T*. *gentile* (**b, d**). **a, b** Semithin sections through the micropylar part; *arrow* thin-walled integumentary cells; *en* endothelium, *es* embryo sac, *mc* micropylar canal, *s* synergid, *star* layers of thick-walled integumentary cells. **c, d** Semithin sections through the chalazal region of the ovule; *arrow* cuticle, *ep* outer epidermis of the integument, *ov* ovary wall, *star* group of compactly arranged small cells. *Bars* = 20 μm

The ovule, at the mature female gametophyte stage, has a considerably thick, multilayer integument, with a heterogeneous structure (Fig. 3a), as occurs in the ovary wall. The female gametophyte is surrounded by a layer of endothelium which differentiates from the inner epidermal cells of the integument (Fig. 3b). A dense cytoplasm of radially stretched endothelial cells contains an irregularly shaped nucleus and numerous dictyosomes, plastids and mitochondria (not shown). The cell walls adjacent to the embryo sac are distinctly thickened and there is no plasmodesmal connection between the endothelium and the central cell of the embryo sac (Fig. 3b). However, there are wall ingrowths on the wall of the central cell adjacent to the endothelium cells (Fig. 3b). The cell walls of the integumentary layers next to the endothelium are extremely thick (Figs. 1b,c and 3b) and PAS positive (Figs. 3a and 4b). The middle lamella is clearly visible as an electron-dense layer between the waving, thick-walled cells, and these special walls have an open, spongy structure (Fig. 3c, d). Due to the gradual excessive thickening of the walls, the cells’ lumen is reduced considerably (Fig. 3b–d). Alterations of the cell walls and a progressive degeneration of the cells surrounding the endothelium take place especially around the chalazal and the central part of the embryo sac (Fig. 3a). The integumentary cells around the micropylar region have thin walls (Figs. 3a and 4a,b), and the cells in direct contact with the micropylar canal are distinguished by a high metabolic activity. The centrally located nucleus and a few small vacuoles are seen in their dense cytoplasm, which is rich in dictyosomes, vesicles differing in size, profiles of rough endoplasmic reticulum, mitochondria and plastids (Fig. 3e). Among the ovules of the species investigated, some differences in the shape of the micropylar canal can be observed. It is more elongated and narrow in the sexually reproducing *T*. *linearisquameum*, whereas in the apomictic *T*. *gentile* it is wide but shorter (Fig. 4a, b). In both species, the ovule’s micropylar canal is filled with an extracellular matrix which reacts positively to PAS (Figs. 3a and 4b). There is also no special deposition of wall material in the integumentary cells situated on the outside of the thick-walled zone. The outer layers of the integument are composed of thin-walled, vacuolated cells (Fig. 3a, f). At the chalazal pole of ovules of both species studied, a group of compactly arranged and distinctly smaller cells stands out just below the epidermis (Fig. 4c, d). Some of these cells have a dense cytoplasm and possess large nuclei.

## Discussion

Studies on *Arabidopsis* ovule mutants in recent years have clearly indicated crosscommunication between the genetically distinct gametophyte and the surrounding sporophytic tissues. Moreover, the studies point out that the formation of viable angiosperm seeds is also strongly dependent on the proper interactions between the seed coat, the embryo and the endosperm (for a review, see Ingram 2010; Bencivenga et al. 2011). Although apomixis is common within Asteraceae (Noyes 2007), the relationships between the sporophytic ovule tissue and the initiation of the diplosporous or aposprous embryo sac formation are poorly recognised. However, some progress has already been made, e.g., in *Hieracium* (Asteraceae), where it has been demonstrated that the initiation and progression of apomictic processes is regulated by funiculus growth and auxin transport (Koltunov et al. 2001; Tucker et al. 2012). Our comparative study of the anatomy of ovules in the sexually reproducing dandelion *T*. *linearisquameum* and the apomictic *T*. *gentile* revealed no essential differences in their structure. The occurrence of a characteristic zonal differentiation of the integument with well-defined layers of thick-walled cells surrounding the endothelium is not dependent on the ploidy level and, at the same time, on the mode of reproduction. Koltunov et al. (1998) observed another type of changes involving an intensive liquefaction of the integument cells closest to the endothelium in *Hieracium* ovules, but this liquefied inner core occurred in both sexual and apomictic plants. A similar pattern of wall modifications as the one in *Taraxacum* has been described in the inner layers of the integument in *Helianthus annuus* (Newcomb 1973a, b), *Bellis perennis* (Engell and Petersen 1977) and two apomictic species of *Chondrilla* (our interpretation of micrographs from Kościńska-Pająk 2006). On the other hand, in the ovules of *Cynara cardunculus*, in the vicinity of the endothelium, the walls of the integument cells remained thin; whereas, thick-walled cells were observed only in the chalazal region of the embryo sac, where specialised nucellar layers formed a hypostase around the podium (Figueiredo et al. 2006). It is also worth mentioning that during the analysis of developmental processes in *Rudbeckia bicolor*, from Asteraceae (Musiał et al. 2012), we observed neither special deposition of cell wall material in the inner layers of the integument nor a liquefied zone surrounding the integumentary tapetum (Musiał, unpublished data). Thus, the examples of *C*. *cardunculus* and *R. bicolor* show that such a special differentiation of the integument is not specific to all the members of Asteraceae and broader comparative studies on the anatomy of ovules are necessary to determine whether this may be a feature of taxonomic importance. According to Anderberg et al. (2007), within the subfamily Cichorioideae, *Taraxacum*, *Chondrilla* and *Hieracium* are included in the tribe Cichorieae, but in different subtribes, Crepidinae and Hieraciinae, respectively. Likewise, *Helianthus* and *Rudbeckia*, representing the subfamily Asteroideae, tribe Heliantheae, have differences at the level of subtribe, namely Helianthinae and Rudbeckiinae. These data suggest that the pattern of anatomical structure of the integument might be useful in Asteraceae classification at the subtribe level, but, again, more extensive studies are required.

Concerning ultrastructure, the thick walls in the dandelion integument cells resemble a mosaic structure alternating with electron-dense bands, which has previously been reported in the epidermal cell walls of mucilaginous leaves, e.g., in *Spartocytisus filipes* and in members of the *Passerina* genus (Lyshede 1977; Bredenkamp and van Wyk 1999) or in the mucilage cells of *Araucaria angustifolia* mesophyll (Mastroberti and de Araujo Mariath 2008). The positive result of PAS reaction indicates that the deposited wall material is rich in water insoluble polysaccharides with 1,2-glycol groups, e.g., pectins. It may possibly provide the necessary nutrients for the proper nourishment of a mature female gametophyte and then of a proembryo. Prominent thick cell walls rich in pectins are typical of the special nutritive tissue that occurs in the ovules or placenta of species of *Utricularia* and *Genlisea* (Płachno and Świątek 2008, 2009). Furthermore, in the case of *Hieracium*, Koltunov et al. (1998) considered that the accumulation of a large pool of nutrients around the embryo sac may favour the evolution of the apomictic trait within the genus. In *Taraxacum*, further investigation is needed to elucidate whether the integument layers adjacent to the endothelium are involved in seed coat differentiation or whether this wall material dissipates during seed development as was reported for *Hieracium* (Koltunov et al. 1998). Some changes in the dandelion integument cells (reduction of protoplast size, enlargement of extracellular matrix) resemble programmed cell death (PCD); however, future detailed ultrastructural studies and also TUNEL reaction should be done to prove this. PCD occurs in plants at all stages of the life cycle. Well known examples of PCD in plants are reproductive tissue, e.g., tapetal cells, nucellar cells, non-functional megaspores, synergids (e.g., Papini et al. 1999, 2011; Fiordi et al. 2002; Brighigna et al. 2006).

This is the first structural description of dandelion ovules based on observations with light and electron microscopy. Although both species studied differ in the ploidy level and the mode of reproduction, their ovules do not show significant differences in their anatomical structure. The special feature of the dandelion ovule is the presence of thick-walled cells in the inner integumentary layers adjacent to the endothelium. Further studies on the anatomy of ovules are required to explain whether this may be a feature of taxonomic importance. A comparison of ovule anatomy in other species may provide useful data for both the taxonomical and phylogenetical investigations not only within the subfamily Cichorioideae but also for the entire Asteraceae.
